# Integrin β3-PKM2 pathway-mediated aerobic glycolysis contributes to mechanical ventilation-induced pulmonary fibrosis

**DOI:** 10.7150/thno.72328

**Published:** 2022-08-15

**Authors:** Shuya Mei, Qiaoyi Xu, Yue Hu, Ri Tang, Jinhua Feng, Yang Zhou, Shunpeng Xing, Yuan Gao, Zhengyu He

**Affiliations:** Department of Critical Care Medicine, Renji Hospital, School of Medicine, Shanghai Jiaotong University, 200127 Shanghai, China.

**Keywords:** integrin β3, pyruvate kinase M2, aerobic glycolysis, mechanical ventilation, pulmonary fibrosis

## Abstract

**Background:** Mechanical ventilation (MV) can induce pulmonary fibrosis. This study aims to investigate whether MV-induced pulmonary fibrosis is associated with aerobic glycolysis and seeks to uncover the underlying mechanisms mediated by integrin β3-pyruvate kinase M2 (PKM2) pathway.

**Methods:** PKM2 knockdown or inhibition, integrin β3 knockout or inhibition and wild-type mice were exposed to MV (20 mL/kg) for 2 h.

**Results:** Mice exposed to MV exhibited increased expression of collagen deposition, and upregulation of α-smooth muscle actin and collagen I in lung tissues. Single cells analysis showed that MV-induced pulmonary fibrosis was associated with increased gene expression of integrin and glycolysis in pulmonary fibroblasts, as well as upregulation of glycolytic products tested by metabolomics. Meanwhile, increased protein level of integrin β3 and PKM2 was confirmed by western blot and immunohistochemistry. Double immunofluorescence staining and flow cytometric analysis showed increased number of fibronectin+/integrin β3+ and fibronectin+/PKM2+ fibroblasts in lung tissues. Furthermore, MV-induced aerobic glycolysis and pulmonary fibrosis were ameliorated after treatment with PKM2 knockdown-AAV and inhibition, or in integrin β3 knockout and inhibition mice.

**Conclusions**: Integrin β3-PKM2 pathway-mediated aerobic glycolysis contributes to MV-induced pulmonary fibrosis. The inhibition of aerobic glycolysis targeting integrin β3-PKM2 pathway may be a promising treatment for MV-induced pulmonary fibrosis.

## Introduction

Pulmonary fibrosis has become one of the most common causes of death in prolonged acute respiratory distress syndrome (ARDS). Most ARDS survivors experience impaired health-related quality of life for years after the acute period, mostly because they develop a fibroproliferative response characterized by fibroblast accumulation and deposition of collagen in the lung 1.

Mechanical ventilation (MV) is the most important supportive therapy for ARDS patients. Despite low tidal volume ventilation is widely promoted and applied, cyclic mechanical stress and mechanical strain may be the cause of ventilation-induced pulmonary injury (VILI) 2-4 and MV-induced pulmonary fibrosis 5-7. MV was found to be a risk factor for the development of pulmonary fibrosis in ARDS subjects, including coronavirus disease 2019 patients 8, 9. To date, the mechanisms associated with MV-induced pulmonary fibrosis have not been completely understood.

Pulmonary fibrosis is characterized by the imbalance in extracellular matrix homeostasis, favoring excess of collagen deposition. Fibroblasts are the principal cell types to regulate extracellular matrix homeostasis. The activation, proliferation, and persistence of fibroblasts mediates extracellular matrix formation and the process of pulmonary fibrosis. In the prevention and treatment of pulmonary fibrosis, it is important to figure out the mechanism underlying fibroblasts activation. Our single cells analysis indicated that MV-induced pulmonary fibrosis was accompanied by increased gene expression of integrin and glycolysis in pulmonary fibroblast. Aerobic glycolysis promoted the fibroblast activation in renal fibrosis 10. PKM2 is the final key regulator in aerobic glycolysis 11. Integrin β3 was found to play an important role in VILI 12. Whether integrin β3 is essential in mediated aerobic glycolysis or possibly involved in MV-induced pulmonary fibrosis is not clear.

In this study, we constructed a high tidal volume ventilation mice model of MV- induced pulmonary fibrosis and used PKM2 knockdown or inhibition and integrin β3 knockout or inhibition mice to clarify the role of the integrin β3-PKM2 pathway in MV- induced pulmonary fibrosis.

## Materials and Methods

### MV model and animal procedures

The study protocol was approved by the institutional Animal Care and Use Committee of Renji hospital, Shanghai Jiao Tong University School of Medicine, Shanghai, China (Permit number: RJ2020-0625). Male C57BL/6 mice (8-12 weeks) were anesthetized with intraperitoneal injection of Ketamine (200 mg/kg) and Xylazine (10 mg/kg), and randomly divided into 2 groups: sham and MV group. Mice in the sham and MV groups were anesthetized and intubated with 20 G arterial cannula. The small animal ventilator (Ventstar, RWD, China) was used for mechanical ventilation to mice. Mice in the MV group were mechanically ventilated for 2 h using FiO2 0.21, VT: 20 mL/kg and respiratory rate (RR) 70 breaths/min 13, while mice in the sham group breathed spontaneously after intubation (Figure S1A). During the MV process, a thermal blanket was used to keep mice warm. After mechanical ventilation, the mice were resuscitated and extubated. Intratracheal injection of PKM2 knockdown adeno-associated virus (AAV) (Figure S1B) and gastric infusion of PKM2 inhibitor shikonin (Selleck, S8279) (Figure S1C) were administered to inhibit PKM2. Integrin β3 knockout mice were also randomly divided into sham and MV groups (Figure S1D). Cilengitide (Selleck, S6387), as integrin β3 inhibitor, was intraperitoneally administered daily for 3 days before MV, at a dose of 4 mg/kg (Figure S1E) 14. The animals were then sent back to the animal facility with free access to water and food and observed for 7 days after intubation. Upon completion of the experiments, the right lung was snap frozen in liquid nitrogen and stored at -80 °C for protein measurements, and the left lung was fixed in paraformaldehyde for pulmonary histopathology and immunohistochemistry.

### PKM2 inhibition by knockdown AAV transfection and Shikonin

AAVs were purchased from Genechem (Shanghai, China). AAV expressing shPKM2 or vector, was delivered to the mice lungs by intratarsal injection of 40 μl PBS containing 1 × 10^12^ μg per mouse, 4 weeks before intubation (Supplemental Figure 1B).

Shikonin, as PKM2 inhibitor, 10 mg/kg in 100ul corn oil, was administered by intragastric injection once a day for 3 consecutive days before intubation (Figure S1C) 15.

### Single-cell analysis

We used single-cell RNA-Seq to analyze mice pulmonary cells from sham (n = 3) and MV (n = 3) groups. The number and viability of prepared single cells were measured using Rigel S2 (Countstar, China). Single-cell libraries were generated with the Chromium Single Cell 3' V2 Chemistry Library Kit, Gel Bead & Multiplex Kit, and Chip Kit from 10x Genomicsr (Biomarker Technologies, China). Cellular suspensions were loaded on the Chromium Controller (10 x Genomics, Pleasanton) to generate Gel Bead-In-Emulsions (GEMs). Barcoded sequencing libraries were performed using Chromium Single Cell 3' Reagent Kits v3.1 (10 x Genomics), according to the manufacturer's instructions. Following the library preparation, the sequencing was performed with paired-end sequencing of 150nt each end on one lane of NovaSeq 6000 per sample. Raw reads were processed using the 10 x Genomics Cell Ranger pipeline (https://support.10xgenomics.com/single-cell-gene-expression/software/downloads/latest) with the mm10 as the reference. Cell Ranger can cluster the single cells, identify the marker genes of each cluster, and export a matrix with unique molecular identifier (UMI) values of each gene in a single cell. The R software package Seurat (https://satijalab.org/seurat, version 2.2) was used for further analysis. Default parameters were used for most of the Seurat analyses. For the Feature Plot function, max cut off was 0.5. The pseudo time trajectory analysis of iSG cells was performed using Monocle 2 (http://cole-trapnell-lab.github.io/monocle-release/). Details were described in the supplement material (Figure S2, Table S1).

### Metabolomics analysis

Ethanol was added to BALF and pulmonary tissue samples (n = 7-8), and shaken vigorously to inactivate any potential viruses, then dried in a biosafety hood. The dried samples were further treated for metabolomics analysis (Biomarker Technologies, China). Briefly, deactivated samples were extracted by adding 300 mL methanol extraction solution. The mixtures were shaken vigorously for 2 min. Proteins were denatured and precipitated by centrifugation. The supernatants contained metabolites of diverse chemical nature. To ensure the quantity and reliability of metabolite detection, four platforms were performed with non-target metabolomics. Each supernatant was divided into four fractions. Two fractions were used for analysis with two separate reverse-phase/ultra-performance liquid chromatography (RP/UPLC)-MS/MS methods with positive ion-mode electrospray ionization (ESI). One fraction was used for analysis with RP/ UPLC-MS/MS with negative-ion mode ESI, and one for analysis using hydrophilic interaction liquid chromatography (HILIC)/UPLC-MS/MS with negative-ion mode ESI. Each fraction was then dried under nitrogen gas to remove the organic solvent and re-dissolved later in four different reconstitution solvents, compatible with each of the four UPLC-MS/MS methods 16.

### Pulmonary histopathology

The left lung was fixed in 4% PFA and stained with Masson's trichrome for collagen identification. Pulmonary fibrosis was quantitatively evaluated through Ashcroft fibrosis score 17. The lungs' histological examination was performed by a pathologist blinded to the experimental groups. Pulmonary injury was scored using five grades from 0 to 4 (0, normal; 1, light; 2, moderate; 3, strong; 4, intense) using the following 9 reference parameters: microscopic atelectasis, microscopic emphysema, perivascular edema, alveolar edema, congestion, alveolar hemorrhage, perivascular hemorrhage, alveolar and interstitial polymorphonuclear leukocytes infiltration, and hyaline membrane formation 16.

### Immunohistochemistry analysis

The samples were cut into 5 mm sections and placed onto slides. Endogenous peroxidase was quenched by H2O2. Mouse monoclonal integrin β3 (SJ1909/NBP2-67416, Novus, USA) and PKM2 antibody (AF7244, R&D, USA) were added on the sections as primary antibodies. The sections were incubated with polymer for 30 min and developed in DAB solution for 5 min. Then, the sections were counterstained with hematoxylin and mounted with cover slip. The Image-Pro Plus 6.0 software was applied to select the same tan color as a uniform criterion to evaluate all positive photos, and five high-power fields of light microscope was used to obtain the cumulative optical density value.

### Immunofluorescence analysis

Paraffin sections (4 μm) of pulmonary tissues were rehydrated and microwaved in citric acid buffer to retrieve antigens. After incubation with 10% BSA for 1 h, the sections were incubated with primary antibodies against integrin β3 (SJ1909/NBP2-67416, Novus, USA), PKM2 (AF7244, R&D, USA), fibronectin and collagen-I (72026S, CST, USA) at a dilution of 1:100 at 4 °C overnight. After washes, sections were incubated with Alexa Fluor 568-conjugated anti-rabbit IgG (Invitrogen, Carlsbad, CA) for integrin β3, PKM2 and collagen-I, fluorescein isothiocyanate (FITC)-conjugated anti-mouse IgG (Invitrogen) for fibronectin at a dilution of 1:400, at 37 ℃, for 1 h in the dark. Finally, nuclei were counterstained with 4'6-diamidino-2-phenylindole (DAPI) (Sigma-Aldrich, USA). Aipathwell softwell (Servicebio, China) was used to analyze the integrated optical density of the single or double stained fluorescent proteins for the semi-quantitative analysis.

### SDS-polyacrylamide gel electrophoresis and Western blotting

Pulmonary tissue was homogenized with lysis buffer, resolved on 10% SDS-PAGE using a Mini-Protean 3 Electrophoresis Cell (Bio-Rad) at 150 V, 100 mA for 1 h, and transferred to polyvinylidene fluoride membrane (Millipore, HVLP04700). The membrane was washed and blocked with Tris-buffered saline (TBS) containing 0.1% Tween-20 and 10% nonfat dry milk before overnight incubation with appropriate primary antibodies (integrin β3 (SJ1909/NBP2-67416, Novus, USA), PKM2 (AF7244, R&D, USA), collagen-I α-1 (COL1A1, 72026S, CST, USA), α-smooth muscle actin (α-SMA) (CST, 19245), Lactate dehydrogenase (LDHA) (3582, CST, USA) and β-actin (8457, CST, USA)) in blocking buffer at 4 °C. The secondary antibodies were then used with 1:6000 goat anti-mouse IgG-Horseradish Peroxidase (HRP) for PKM2, or 1:6000 goat anti-rabbit IgG HRP for integrin β3, COL1A1, α-SMA and β-actin, in blocking buffer for 1 hour at room temperature. The bands were visualized using enhanced chemiluminescence (Amersham ECL Western Blotting Detection Reagents, GE Healthcare, Baie d'Urfe, QC, Canada).

### Enzyme-linked immunosorbent assay

Bronchoalveolar lavage fluid (BALF) samples were collected using a 20 G intratracheal cannula, washing the right lung one time with 500 μL of cold PBS, and by pooling and centrifuging at 3000 g for 10 min the recovered BALF. The supernatants were stored at -80 °C.

Blood samples were collected from anesthetized animals by cardiac puncture, placed into coagulation-promoting tubes and centrifuged at 3000 g for 10 min. The serum was stored at -80 °C.

The levels of lactate in serum and BALFs were measured with the Lactate Assay Kit (BioVision, K607) according to the manufacturer's instructions. The level of type I procollagen carboxy - terminal peptide (PICP) in BALF was tested with the PICP ELISA Kit (MULTISCIENCES BIOTECH, China).

### Flow cytometry

The whole pulmonary tissue was enzymatically digested using Pulmonary Dissociation Kit (130-095-927, Miltenyi Biotec, USA). The gentle MACS™ Dissociations were used for mechanical dissociation steps. After dissociation, the samples were applied to a 70 µm filter to remove any remaining larger particles from the single-cell suspension. Then, single-cell suspension was incubated with integrin β3 (562917, BD, USA) at room temperature for 10min, after removing red blood cell by Lysing Buffer (555899, BD Pharm Lyse™, USA). After the cell surface staining procedure was completed, cells were Fix/Permed by Transcription Factor Buffer Set (562574, BD, USA) and incubated with PKM2 (NBP1-48308AF488, Novus, USA) and Fibronectin (NBP2-34504AF647, Novus, USA) for 30 min. After washing with PBS, the cells were analyzed by flow cytometry (BD FACS Verse TM, USA); data were analyzed with the Flow jo (version 10) software.

### Statistical analysis

GraphPad Prism 5.0 (GraphPad Software Inc, La Jolla, CA) was used for the analysis. Experiments were done at least in triplicate. Data are reported as mean ± SEM. One-way analysis of variance (ANOVA) was used to determine the means of three or more treatment groups. Student's t-test (two tailed) was used to compare the differences between two groups. Differences were considered statistically significant if *P* < 0.05.

## Results

### MV induces pulmonary fibrosis

**Figure 1:**
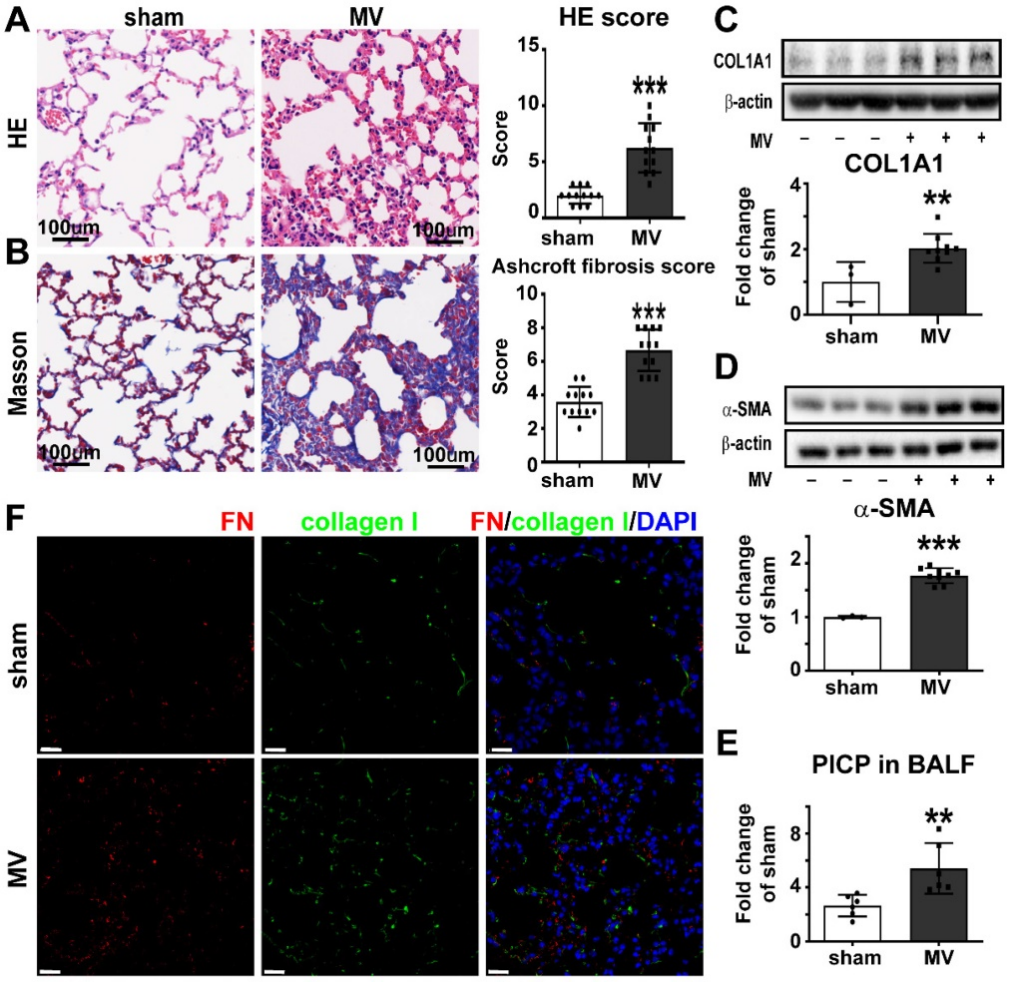
** Mechanical ventilation induces pulmonary fibrosis.** Mice lung tissue were acquired at Day 7 after 2 h of MV.** A**, lung injury was accessed and scored by Hematoxylin and Eosin staining. Original magnification × 200. Scale bars correspond to 100 µm, n = 12 per group. **B**, collagen deposition was assessed with Masson's trichrome staining and evaluated through Ashcroft fibrosis score. Original magnification × 200. Scale bars correspond to 100 µm, n = 12 per group. **C-E**, fibrosis was also quantified by determination of collagen-I α1 (COL1A1) (**C**), α smooth muscle actin (α-SMA) (**D**) in lung tissues (n = 3 in sham group and n = 9 in MV group) and type I procollagen carboxy-terminal peptide (PICP) levels in bronchoalveolar lavage fluid (BALF) (**E,** n = 6 per group). **F**, lung tissues were stained with fluorophore-labeled antibodies against fibroblast marker fibronectin (FN) (Alexa Fluor 568, red) and collagen-I (fluorescein isothiocyanate, green). 4',6-diamidino-2- phenylindole (DAPI) stain was used to detect nuclei (blue). Original magnification × 400, Scale bars correspond to 20 µm. Data are expressed as means ± SEM. * p < 0.05, **p < 0.01, *** p < 0.001 (t test).

To create the MV-induced pulmonary fibrosis model *in vivo*, mice were observed 7 days after 20 mL/kg MV for 2 h. Histologically, pulmonary injury, interstitial leukocytes infiltration, alveolar edema, and hemorrhage were more obvious after MV, if compared to sham group. The total pulmonary injury score increased in the MV group (Figure 1A). Masson staining displayed an increased collagen deposition in the pulmonary interstitium after MV (Figure 1B). This was also associated with thickened alveolar walls and tissue patches (Figure S3A), accompanied with upregulation of collagen-I and α-SMA contents in pulmonary tissue (Figure 1C, D). In addition, increase of PICP levels was observed in BALF after MV (Figure 1E).

Fibroblasts are the major cells responsible for extracellular matrix's fibronectin and collagen synthesis in pulmonary fibrosis. We also investigated the colocalization of fibroblast marker fibronectin (FN) and collagen I in the alveolar septum, as show in Figure 1F. Consistent with the histologic observations, MV significantly increased the percentage of fibronectin+/collagen I+ cells in total cells, compared with the nonventilated control group (Figure S3B).

### MV leads to upregulation of integrin β3 and PKM2 in pulmonary fibroblasts

**Figure 2:**
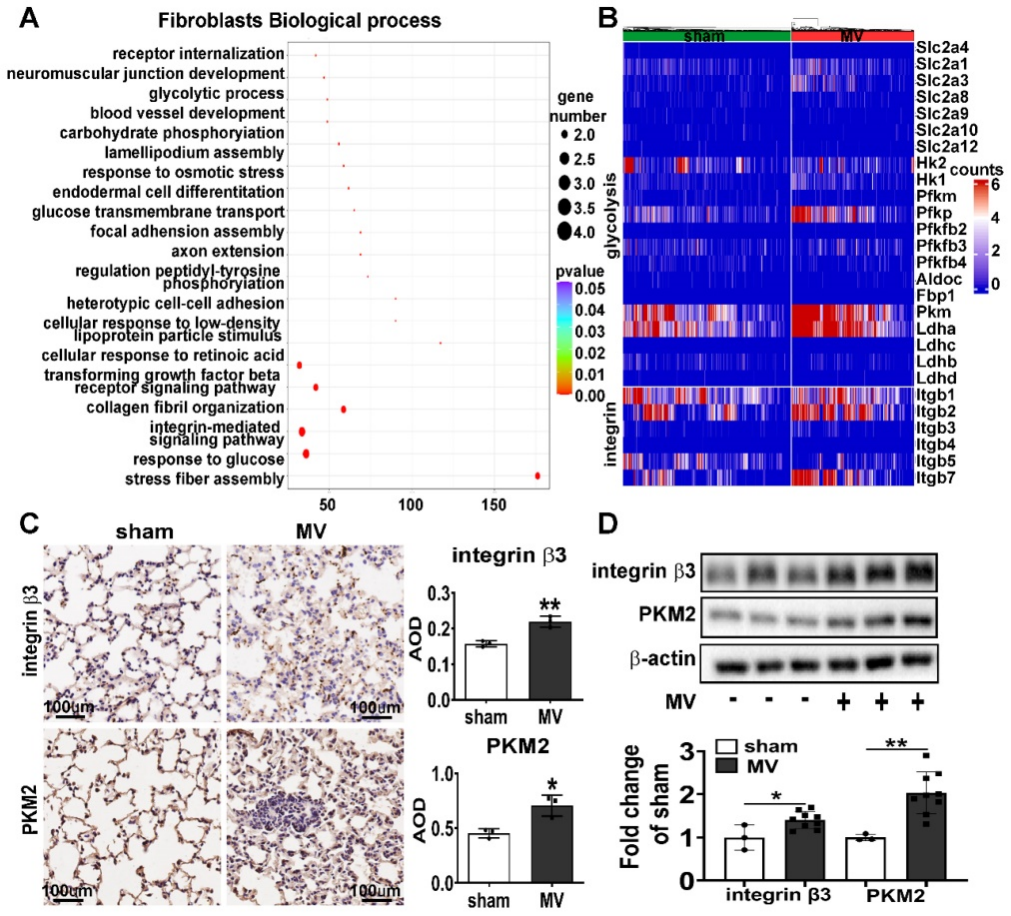
** MV leads to upregulation of integrin β3 and PKM2 in pulmonary fibroblasts.** Mice lung tissue were acquired at Day 7 after 2 h of MV. **A**, functional enrichment analysis with GO Biological Processes was performed using GOrilla with the differential genes upregulated and downregulated in pulmonary fibroblasts cluster. **B**, heatmaps are showing the upregulated and downregulated genes of integrin and glycolysis in pulmonary fibroblasts cluster. **C**, protein expression of integrin β3 and pyruvate kinase M2 (PKM2) in lung homogenates was assessed by immunohistochemistry and quantified by the average optical density value (AOD), n = 3 per group. **D**, protein expression of integrin β3 and PKM2 in lung homogenates was determined by western blot analysis. Relative densitometry of the protein bands of integrin β3 and PKM2 over β-actin is displayed in bar graphs, n = 3 in sham group and n = 9 in MV group. Data are expressed as means ± SEM. *p < 0.05, **p < 0.01, *** p < 0.001(t test).

To further explore the mechanisms associated with MV-induced pulmonary fibrosis, we used single-cell RNA-Seq analysis and found the pathway enrichment analysis on the response to glucose and integrin-mediated signaling pathway were significantly different in pulmonary fibroblasts (Figure 2A). Then we focused on the gene about integrin and glycolysis which has been previously reported 18-22 and found integrin and glycolysis target gene expression of total pulmonary fibroblasts were upregulated after MV (Figure 2B).

**Figure 3:**
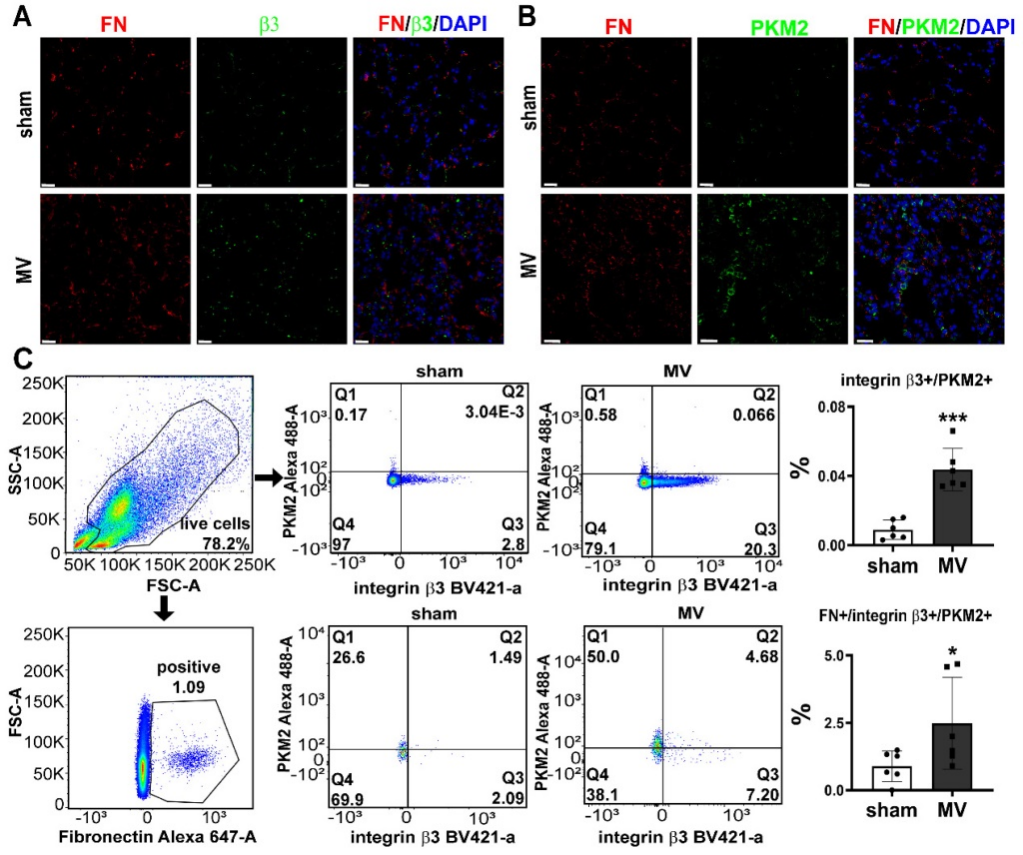
** MV leads to upregulation of integrin β3 and PKM2 in pulmonary fibroblasts.** Mice lung tissue were acquired at Day 7 after 2 h of MV. **A and B,** lung tissues were stained with fluorophore-labeled antibodies against fibroblast marker fibronectin (FN) (Alexa Fluor 568, red), integrin β3(β3) (fluorescein isothiocyanate, green) and PKM2 (fluorescein isothiocyanate, green). 4',6-diamidino-2- phenylindole (DAPI) stain was used to detect nuclei (blue). Original magnification × 400. Scale bars correspond to 20 µm. **C**, flow cytometry was used to detect the expression of integrin β3 and pyruvate kinase M2 (PKM2) in lung cells and in fibronectin (FN) positive fibroblasts, n = 6 per group. Data are expressed as means ± SEM. *p < 0.05, **p < 0.01, *** p < 0.001(t test).

To further validate single-cell RNA-Seq data, we examined the expression of integrin β3 and PKM2 in MV-pulmonary fibrosis lung tissue. Consistent with these findings, upregulated protein level of integrin β3 and PKM2 in pulmonary tissue was detected by IHC and WB analysis (Figure 2C, D). Increased amounts of fibronectin+/integrin β3+ and fibronectin+/PKM2+ fibroblasts were observed in MV group (Figure 3A, B). Flow cytometry was also used to detect the expression of integrin β3, PKM2 and fibronectin in pulmonary cells. The percentage of integrin β3+/PKM2+ cells and integrin β3+/PKM2+/fibronectin+ fibroblasts were all increased significantly after MV (Figure 3C).

All these elements together indicated that the activations of integrin β3 and PKM2 in fibroblasts were involved in MV-induced pulmonary fibrosis.

### Aerobic glycolysis was associated with MV-induced pulmonary fibrosis

**Figure 4:**
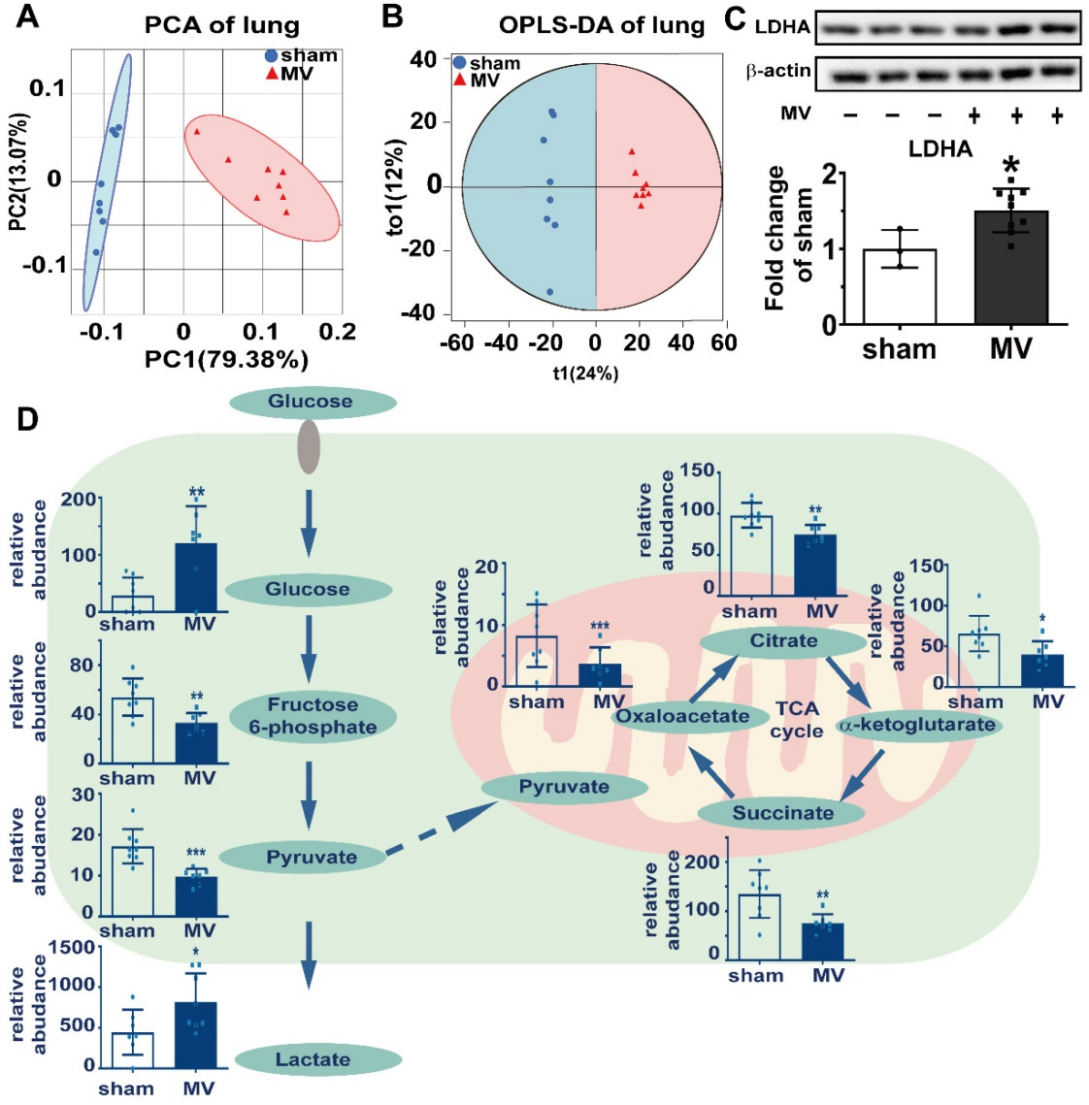
** Aerobic glycolysis was associated with MV-induced pulmonary fibrosis.** Mice lung tissue were acquired at Day 7 after 2 h of MV. **A and B,** consistency and reproducibility of lung tissues analyzed by principle component analysis (PCA) and orthogonal projections to latent structures-discriminant analysis (OPLS-DA), n = 8 per group. **C**, lactic dehydrogenase (LDHA) was quantified by western blot analysis, n = 3 in sham group and n = 9 in MV group. **D**, glucose metabolites were accessed by metabolomics analysis of lung tissues and BALF, n = 7 - 8 respectively. Data are presented as mean ± SEM, *p < 0.05, **p < 0.01, *** p < 0.001 (t test).

To clarify the relationship between aerobic glycolysis and MV-induced pulmonary fibrosis, we used ultra-performance liquid chromatography/tandem mass spectrometry (UPLC-MS/MS) untargeted metabolomics approach to analyze the pulmonary and BALF samples. Our data were acquired with high degree of consistency and reproducibility in pulmonary and BALF samples analyzed by orthogonal projections to latent structures- discriminant analysis (OPLS-DA) (Figure 4A and B, Figure S4). Metabolites of glucose metabolism and tricarboxylic acid cycle, such as fructose 6-phospate, pyruvate, oxalacetate, citrate, α-ketoglutarate and succinate, were enriched in the pulmonary samples of sham group, while metabolites of aerobic glycolysis such as glucose in the pulmonary samples and lactate in BALF samples were more in MV group (Figure 4D). Consistent with these metabolic findings, we also detected increased protein level of LDHA as the key enzyme for aerobic glycolysis (Figure 4C) in pulmonary tissue. These findings are indicating that aerobic glycolysis might mediate MV-induced pulmonary fibrosis.

### PKM2 inhibition alleviated aerobic glycolysis and MV-induced pulmonary fibrosis

**Figure 5:**
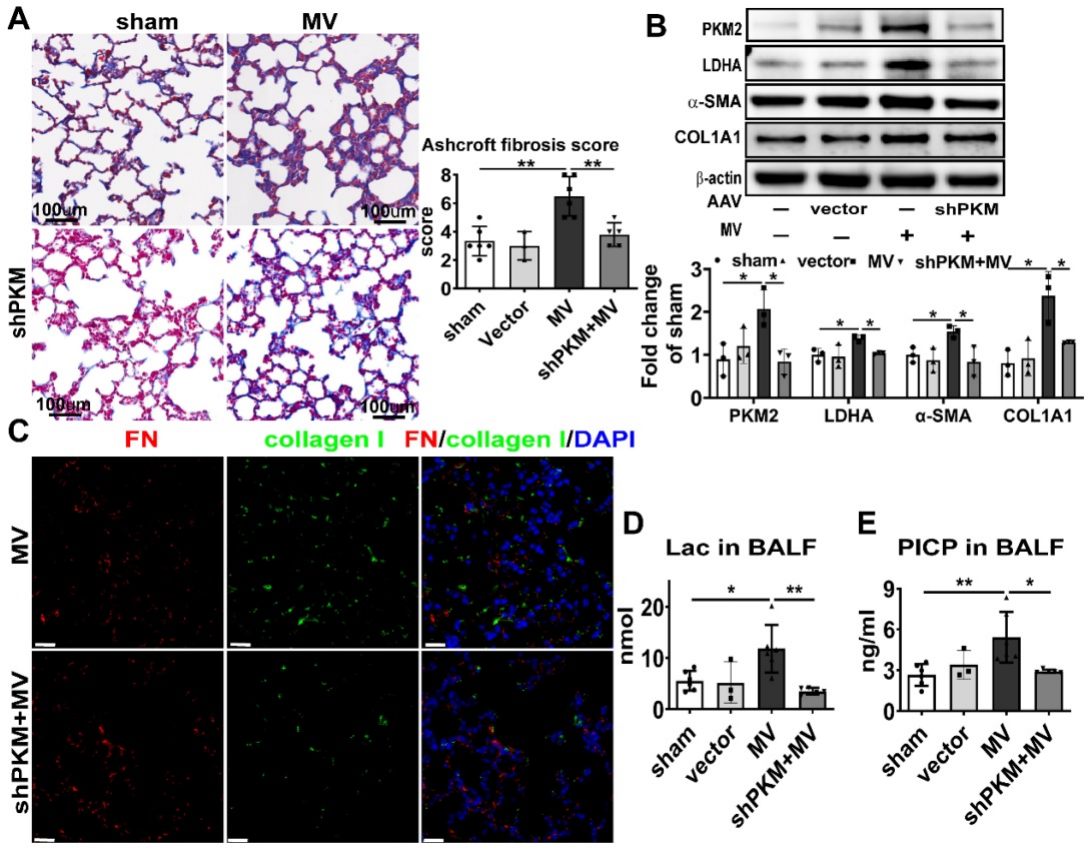
** PKM2 knockdown alleviates aerobic glycolysis and MV-induced pulmonary fibrosis.** Mice were treated intratracheally with vector-AAV or shPKM-AAV 4 weeks before mechanical ventilation (MV), for the duration of 2 h. Lung tissues were harvested 7 days after intubation. **A**, lung fibrosis was quantified by Masson's trichrome staining. Original magnification × 200, Scale bars correspond to 100 µm, n = 6, 3, 6, 5, respectively in each group. **B**, pyruvate kinase M2 (PKM2), lactic dehydrogenase (LDHA), α smooth muscle actin (α-SMA) and collagen I α1 (COL1A1) expression was quantified by western blot analysis, n = 3 per group. **C**, lung tissues were stained with fluorophore labeled antibodies against fibroblast marker fibronectin (FN) (red) and collagen I (green), 4',6-diamidino-2- phenylindole (DAPI) stain was used to detect nuclei (blue), Original magnification × 400, Scale bars correspond to 20 µm. **D** and **E**, lactate (Lac) and type I procollagen carboxy-terminal peptide (PICP) in bronchoalveolar lavage fluid (BALF) was tested, n = 6, 3, 6, 5, respectively in each group. Data are expressed as means ± SEM, *p < 0.05, **p < 0.01, *** p < 0.001 (ANOVA).

PKM2 is the final key enzyme regulating aerobic glycolysis, and its expression and activation are directly associated with the beginning of aerobic glycolysis 11. To determine the effect of PKM2 on aerobic glycolysis and MV induced-pulmonary fibrosis, shPKM2-AAV was transfected by intratracheal injection to knockdown the PKM2 gene in the mouse pulmonary tissue with the decrease of PKM2 after MV, as shown in Figure 5B. PKM2 downregulation precluded the increase of LDHA in pulmonary tissue and lactate and PICP in BALF (Figure 5B, D, E), accompanied with decrease of the collagen deposition (Figure 5A, Figure S5A), collagen-I and α-SMA downregulation (Figure 5B) in pulmonary tissue induced by MV, and followed by a decrease of fibronectin and collagen-I dual positive cells in the pulmonary tissue (Figure 5C, Figure S5B).

**Figure 6:**
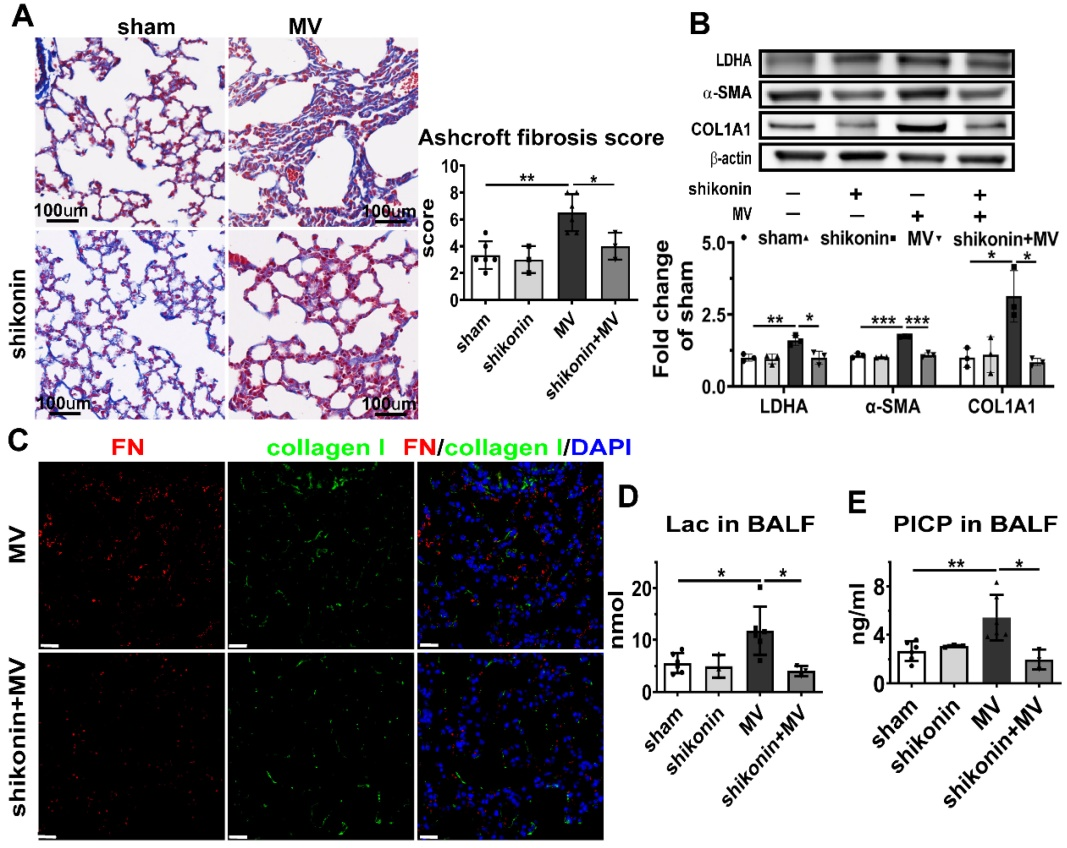
** PKM2 inhibitor alleviates aerobic glycolysis and MV-induced pulmonary fibrosis.** Mice were treated with PKM2 inhibitor shikonin 10 mg/kg in 100ul corn oil daily for 3 consecutive days before being subjected to MV for 2 h. Lung tissues were harvested 7 days after intubation. **A**, lung fibrosis was accessed by Masson's trichrome staining, Original magnification × 200, Scale bars correspond to 100 µm, n = 6, 3, 6, 3, respectively in each group. **B**, lactic dehydrogenase (LDHA), α smooth muscle actin (α-SMA) and collagen I α1 (COL1A1) expression was quantified by western blot analysis, n = 3 per group. **C**, lung tissues were stained with fluorophore-labeled antibodies against fibroblast marker fibronectin (FN) (red) and collagen I (green), 4',6-diamidino-2- phenylindole (DAPI) stain was used to detect nuclei (blue), original magnification × 400, Scale bars correspond to 20 µm. **D** and **E**, lactate (Lac) and type I procollagen carboxy-terminal peptide (PICP) levels in BALF was tested, n = 6, 3, 6, 3, respectively in each group. Data are expressed as means ± SEM, *p < 0.05, **p < 0.01, *** p < 0.001 (ANOVA).

To further investigate whether pharmacologically altering PKM2 activation *in vivo* could affect MV-induced pulmonary fibrosis, PKM2 in the mouse pulmonary tissue was inhibited by gastric infusion of shikonin. Similar to the effect of PKM2 knockdown, shikonin treatment precluded MV-induced elevated lactate and PICP production in BALF (Figure 6D, E), collagen deposition (Figure 6A, Figure S6A), LDHA, α-SMA and collagen I contents (Figure 6B) in pulmonary tissue, along with a decrease of fibronectin and collagen-I dual positive cells (Figure 6C, Figure S6B).

These findings are suggesting that MV activates PKM2 to accelerate aerobic glycolysis and to induce pulmonary fibrosis. Therefore, the inhibition of PKM2 precluded this process.

### Integrin β3 downregulation ameliorated PKM2-mediated aerobic glycolysis and MV-induced pulmonary fibrosis

**Figure 7:**
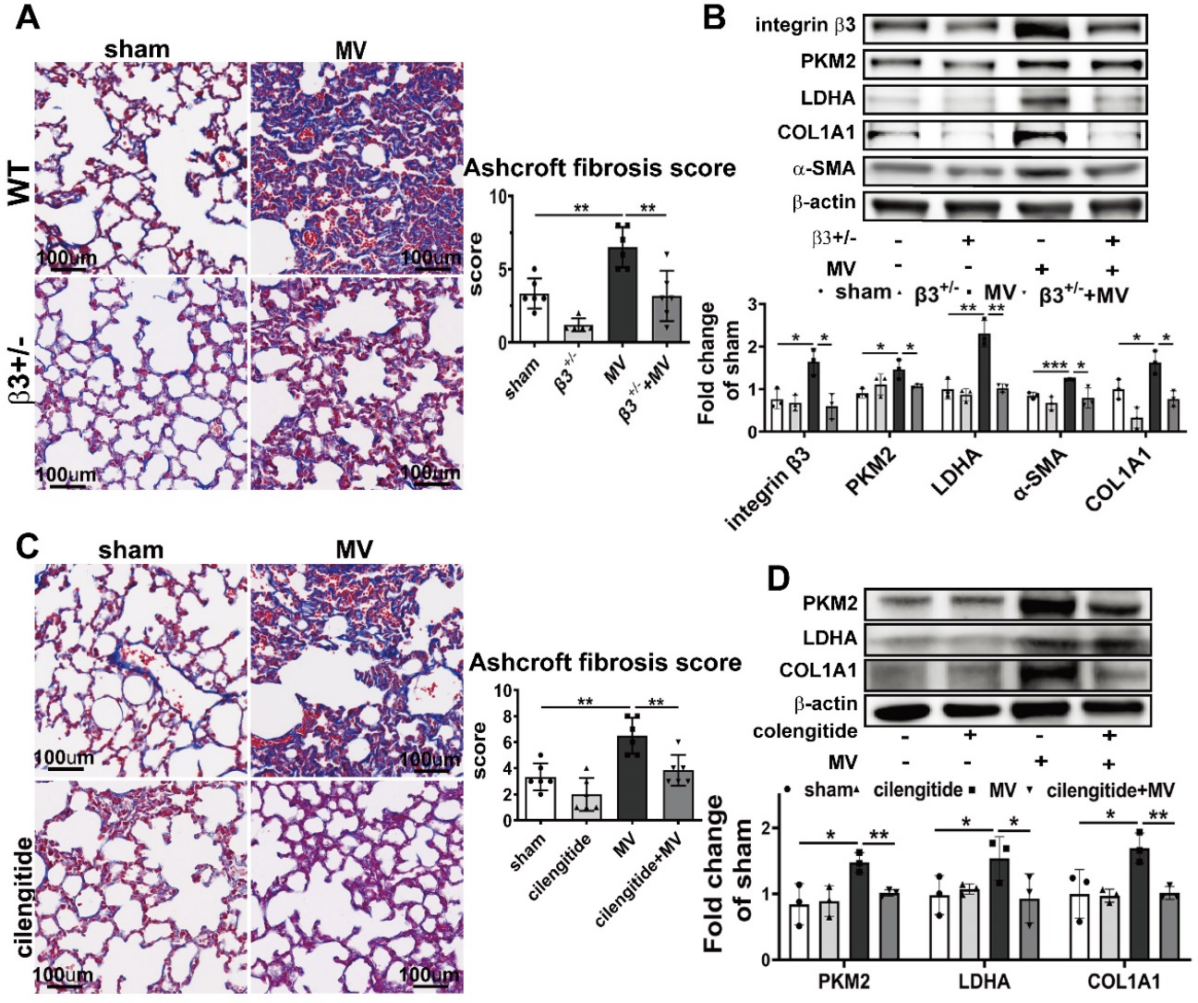
** Integrin β3 inhibition ameliorates PKM2-mediated aerobic glycolysis and MV-induced pulmonary fibrosis. A** and **B**, both wild-type and integrin β3-deficient (β3^+/-^) mice were subjected to mechanical ventilation (MV) for 2 h. Lung tissues were harvested at day 7. **A**, lung fibrosis was quantified by Masson's trichrome staining, original magnification × 200, scale bars correspond to 100μm, n = 6, 5, 6, 6, respectively in each group. **B**, integrin β3, pyruvate kinase M2 (PKM2), lactic dehydrogenase (LDHA), collagen I α1 (COL1A1) and α smooth muscle actin (α-SMA) expression was quantified by western blot analysis, n = 3 per group.** C** and **D**, mice were treated intraperitoneally with integrin β3 inhibitor cilengitide 4 mg/kg once a day for 3 consecutive days before being subjected to MV for 2 h. Lung tissues were harvested 7 days after intubation. **C**, lung fibrosis was quantified by Masson's trichrome staining, original magnification × 200, scale bars correspond to 100 µm, n = 6 per group. **D**, integrin β3, PKM2, LDHA and COL1A1 expression was quantified by western blot analysis, n = 3 per group. Data are expressed as means ± SEM, *p < 0.05, **p < 0.01, *** p < 0.001 (ANOVA).

**Figure 8:**
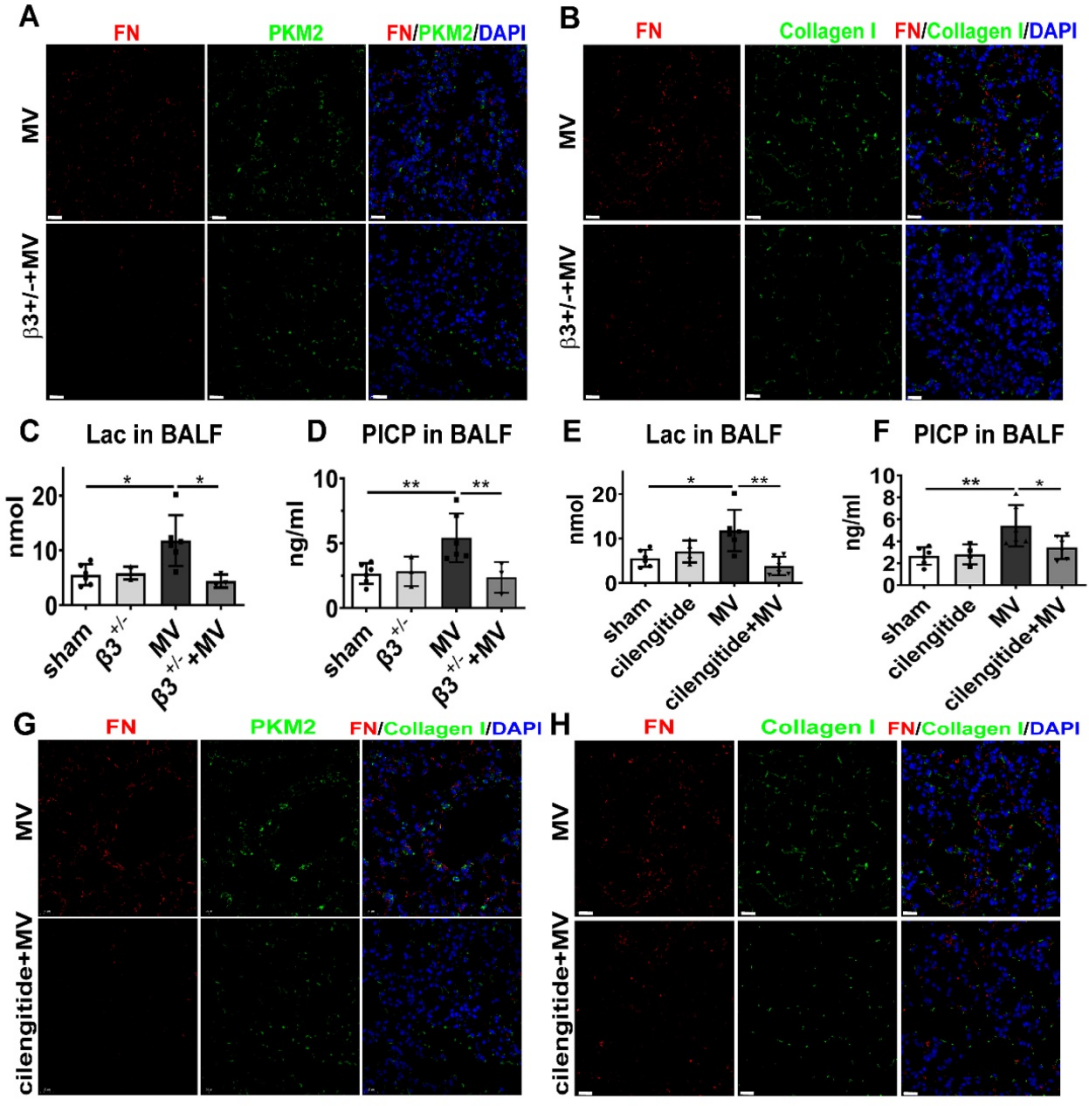
** Integrin β3 inhibition ameliorates PKM2-mediated aerobic glycolysis and MV-induced pulmonary fibrosis**. **A-D**, both wild-type and integrin β3-deficient (β3^+/-^) mice were subjected to mechanical ventilation (MV) for 2 h. Lung tissues were harvested at day 7. **A** and **B**, lung tissues were stained with fluorophorelabeled antibodies against fibroblast marker fibronectin (FN) (red), pyruvate kinase M2 (PKM2) (green) and collagen I (green), 4',6-diamidino-2- phenylindole (DAPI) stain was used to detect nuclei (blue), original magnification × 400, scale bars correspond to 20 µm. **C** and **D**, lactate (Lac) and type I procollagen carboxyterminal peptide (PICP) levels in bronchoalveolar lavage fluid (BALF) were tested, n = 6, 3, 6, 3, respectively in each group. **E-H**, mice were treated intraperitoneally with integrin β3 inhibitor cilengitide 4mg/kg once a day for 3 consecutive days before being subjected to MV for 2h. Lung tissues were harvested 7days after intubation. **E** and **F**, Lac and PICP were tested in BALF, n = 6, 4, 6, 6, respectively in each group. **G** and **H**, lung tissues were stained with fluorophore-labeled antibodies against fibroblast marker FN (red), PKM2 (green) and collagen I (green), DAPI stain was used to detect nuclei (blue), original magnification × 400, scale bars correspond to 20 µm. Data are expressed as means ± SEM, *p < 0.05, **p < 0.01, *** p < 0.001 (ANOVA).

To assess whether integrin β3 is essential to MV-induced PKM2 activation and pulmonary fibrosis, we examined the PKM2 expression and aerobic glycolysis in integrin β3-deficient mice, 7 days after MV. As expected, western blot showed that integrin β3 protein expression in pulmonary tissue was decreased dramatically in integrin β3-deficient mice (Figure 7B). Meanwhile, downregulation of PKM2 was also detected by WB (Figure 7B), and it was further confirmed by immunofluorescence (Figure 8A, Figure S8A), together with a decreased level of LDHA in lung (Figure 7B) and lactate in BALF (Figure 8A). MV-induced pulmonary fibrosis in integrin β3-deficient mice was alleviated by downregulating collagen deposition (Figure 7A, Figure S7A), collagen I and α-SMA contents (Figure 7B), fibronectin+/collagen+ fibroblasts in pulmonary tissue (Figure 8B, Figure S8B) and PICP in BALF (Figure 8D).

To further investigate whether pharmacologically altering integrin β3 activation *in vivo* could affect MV-induced pulmonary fibrosis, integrin β3 was inhibited in the mouse pulmonary tissue by intraperitoneal injection of cilengitide. Similar to the effect in integrin β3-deficient mice, cilengitide treatment in MV group downregulated PKM2 and LDHA expression in lung (Figure 7D, 8G, Figure S8C) and lactate production in BALF (Figure 8E), along with a decrease of collagen deposition (Figure 7C, Figure S7B), collagen I contents (Figure 7D), fibronectin/collagen-I dual positive fibroblasts in pulmonary tissue (Figure 8H, Figure S8D) and PICP in BALF (Figure 8F).

Therefore, we speculated that MV activated integrin β3-PKM2 pathway to elevate aerobic glycolysis and to mediate pulmonary fibrosis.

## Discussion

Mechanical ventilation (MV), as an effective treatment for respiratory failure, can also induce VILI and pulmonary fibrosis, and this limits its widespread use in clinical practice 23, 24. Rodent models exposed to high-tidal-volume ventilation have been used to investigate the pathomechanisms of MV-induced pulmonary fibrosis 25, 26, and our results were consistent with these investigations. Nevertheless, the mechanisms underlying MV-induced pulmonary fibrosis are still unclear. Our study clarified the role of integrin β3-PKM2 regulatory pathway in MV-induced pulmonary fibrosis.

Aerobic glycolysis has been increasingly recognized as an important pathogenic process that underlies many types of fibrosis 21. But the mechanism that regulates aerobic glycolysis in MV-induced pulmonary fibrosis has not been investigated. PKM2 is the final rate-limiting kinase in aerobic glycolysis 11. PKM2-dependent aerobic glycolysis was found participating in the regulation of heart, kidney, liver and many other organ fibrosis 27-29. In our previous studies we found that aerobic glycolysis of pulmonary fibroblast was involved in lipopolysaccharide-induced pulmonary fibrosis 30. Consistent with these results, this study revealed that the inhibition of PKM2 by knockdown-AAV and inhibitor shikonin alleviated the aerobic glycolysis and pulmonary fibrosis induced by MV. Finally suggesting that PKM2-dependent aerobic glycolysis could be involved in the regulation of MV-induced pulmonary fibrosis.

Resolving the metabolic alterations of PKM2-dependent aerobic glycolysis may provide a novel strategy for antifibrosis therapy. Shikonin is one of the active principles of Zicao, the root extract of Lithospermum erythrorhizon, widely used in traditional Chinese medicine and approved in Japan as food dye 15. Shikonin is also a potent and specific PKM2 inhibitor 21. It has been demonstrated that shikonin has interesting pharmacological properties, including anti-inflammatory, anticancer, antimicrobial, and wound healing effects 15, also proven to be safe in clinical trials 31, 32. Furthermore, shikonin has the additional effect of antagonizing hepatic, renal, skin and other tissue fibrosis 32-34. Shikonin might serve as a therapeutically candidate for MV-induced pulmonary fibrosis.

Integrins are heterodimeric cell adhesion molecules which are traditionally considered to maintain the mechanical connection between cells and the extracellular matrix 35-37. In addition to the anchorage of cells to the extracellular matrix, integrins have critical functions in intracellular signaling and take center stage in many physiological and pathological conditions 18, 19, 38. Recent studies are indicating that integrins could represent biochemical and mechanical sensors to respond to and interact with ECM of differing properties 38, 39. Ding X et al. 40 and Kelly GT et al. 41 found that integrins are critical mechanical sensors to mediate VILI. In our previous study we found that integrin β3 upregulation was involved in lipopolysaccharide -induced pulmonary fibrosis 42. It has been reported that integrins' activation could further activate PKM2-dependent aerobic glycolysis in cancers 43-45. In this study, we found that integrin β3 was involved in MV-induced pulmonary fibrosis, along with the enhanced PKM2-dependent aerobic glycolysis. Inhibition of integrin β3 mitigated PKM2-dependent aerobic glycolysis and pulmonary fibrosis induced by MV, which indicated integrin β3 as a mechanical sensor accelerated PKM2-dependent aerobic glycolysis to induce pulmonary fibrosis.

Integrins-targeted inhibitors represent a very active area of research and development in the fibrosis field, including phase 2 trials of inhibitors of αvβ6 (NCT01371305), αvβ1 and αvβ6 (NCT04072315), and αvβ1, αvβ3 and αvβ6 (NCT03949530) in pulmonary fibrosis. Cilengitide, as αvβ3 and αvβ5 integrin antagonist, displayed an encouraging activity in Phase II clinical trials and is currently being tested in a Phase III trial in patients with glioblastoma (NCT00689221). Nevertheless, there are currently no evidence about cilengitide being applied in pulmonary fibrosis. This study suggests that cilengitide could be considered for clinical treatment in MV-induced pulmonary fibrosis.

## Conclusion

In summary, this study characterizes integrin β3 as a mechanical sensor upregulated PKM2 to accelerate aerobic glycolysis and induce pulmonary fibrosis by MV. As a result, mechanical microenvironment and metabolically targeted therapies could become perspective strategies in MV-induced pulmonary fibrosis.

## Supplementary Material

Supplementary materials and methods, figures and table.

## Abbreviations

MV: mechanical ventilation
PKM2: pyruvate kinase M2
ARDS: acute respiratory distress syndrome
VILI: ventilation-induced pulmonary injury
α-SMA: α smooth muscle actin
PICP: type I procollagen carboxy-terminal peptide
BALF: bronchoalveolar lavage fluid
FN: fibronectin
COL1A1: collagen-I α1
DAPI: 4',6-diamidino-2- phenylindole
AOD: average optical density value
