# Supplementary material for: Integrin β3-PKM2 pathway-mediated aerobic glycolysis contributes to mechanical ventilation-induced pulmonary fibrosis
Source: Theranostics. 2022 Aug 15;12(14):6057–68. doi: 10.7150/thno.72328 (PMC9475464; doi:10.7150/thno.72328)
Supplement: Supplementary file 1 — Supplementary materials and methods, figures and table. [file thnov12p6057s1.pdf]

Supplemental Materials

Materials and Methods

Single-cell analysis

Totally, 44,975 cells were sequenced. The CellRanger analysis of cell information statistics were indicated in the supplemental table1. The minimum number of genes in cells was generally 500. The filtering parameters of this project were set as: (1) the number of cells with gene expression: ≥ 10; (2) mitochondrial content (%): ≤ 20; (3) UMI Number: ≥ 100; (4) Number of genes: 500 ~ 7000. The number of pulmonary fibroblasts shown in Figure 2B was 9,619. Cell clustering analysis was based on the identification of marker genes, and the marker genes of the subgroup obtained by clustering were compared with the marker genes of known cell types in the database. SingleR was used to automatically annotate cell populations. SingleR was a software for automatic annotation of scRNA-seq data based on Spearman correlation. Enrichment was used to perform gene set enrichment analysis against the Gene Ontology Biological Process 2018 version gene set collection. The MSigDB Hallmark was also used for the gene sets, for which Fisher's exact test was used to compute the enrichment scores. In both cases, we corrected for multiple hypothesis testing using the Benjamini-Hochberg procedure. Before clustering cells, Uniform Manifold Approximation and Projection (UMAP) was used to reduce the dimensionality of the data (Figure **S**2).

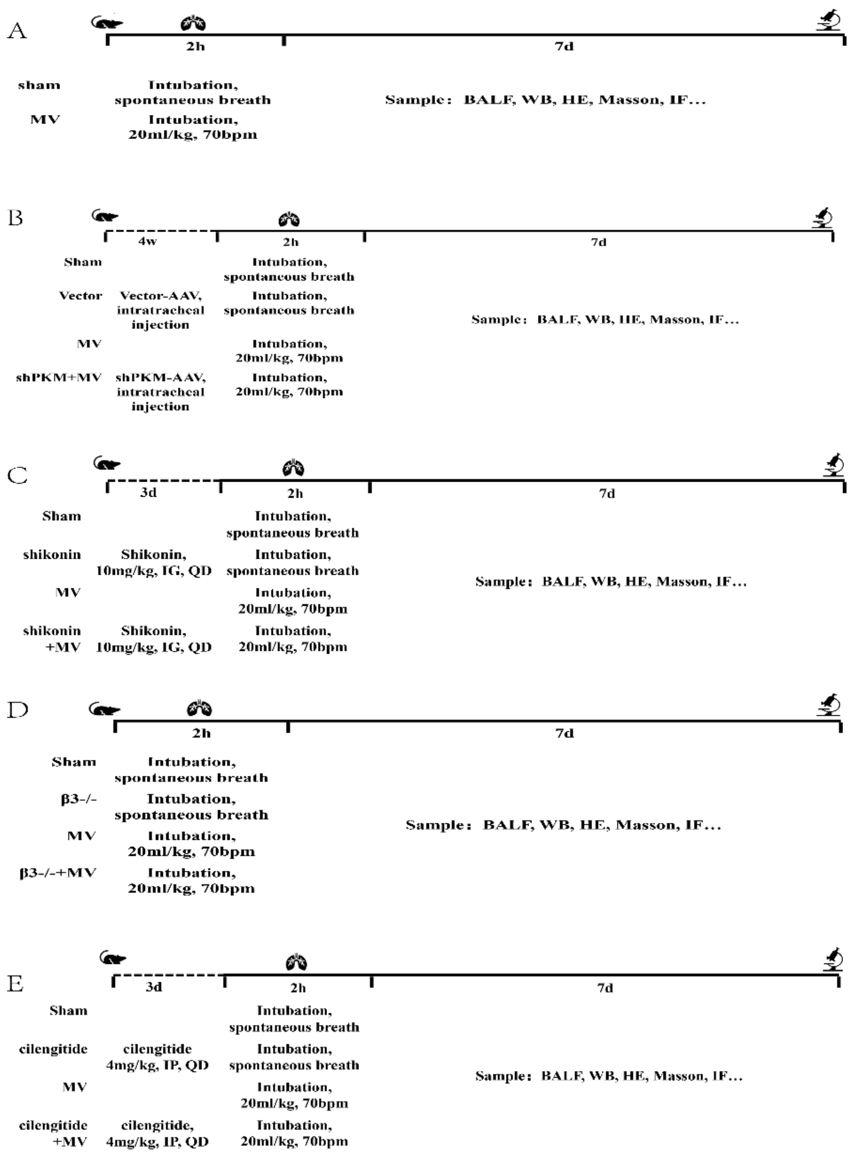

Figure S1. MV model and animal procedures. **A**, mice in the MV group were mechanically ventilated for 2 h using FiO2 0.21, VT: 20 ml/kg and respiratory rate 70 bpm, while mice in the sham group breathed spontaneously after intubation. **B**, intratracheal injection of PKM2 knockdown adeno-associated virus (AAV) and vector-AAV. **C**, gastric infusion of PKM2 inhibitor shikonin 10 mg/kg were administered to inhibit PKM2 daily for 3 days before MV. **D**, Integrin β3 knockout mice were randomly divided into sham and MV groups. **E**, Cilengitide, as integrin β3 inhibitor, was intraperitoneally administered daily for 3 days before MV, at a dose of 4 mg/kg.

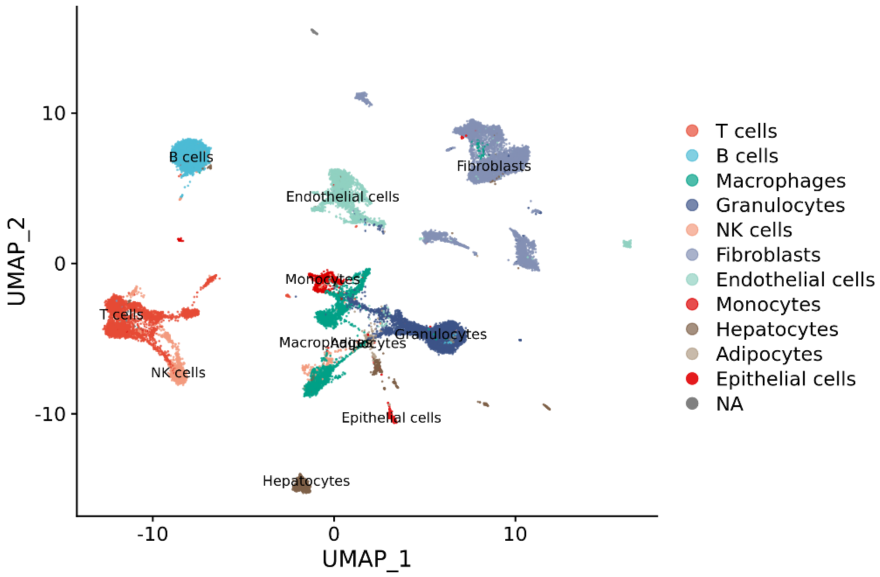

Figure S2. Cell type identification UMAP plot. Cell clustering analysis was based on the identification of marker genes. Uniform Manifold Approximation and Projection (UMAP) was used to reduce the dimensionality of the data.

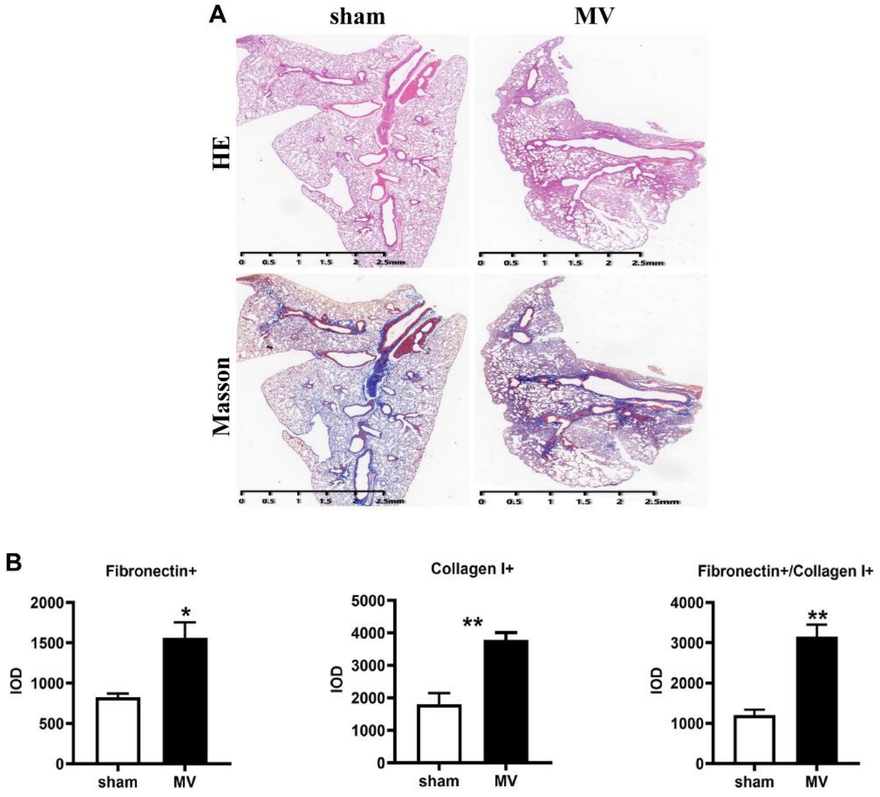

Figure S3. Mechanical ventilation induces pulmonary fibrosis. Mice lung tissue were acquired at Day 7 after 2 h of MV. A, lung injury was accessed and scored by Hematoxylin and Eosin staining.

Original magnification × 10. Scale bars correspond to 2.5 mm, n = 12 per group. B, collagen deposition was assessed with Masson's trichrome staining and evaluated through Ashcroft fibrosis score. Original magnification × 10. Scale bars correspond to 2.5 mm, n = 12 per group. **B**, lung tissues were stained with fluorophore-labeled antibodies against fibroblast marker fibronectin and collagen-I. 4',6-diamidino-2- phenylindole (DAPI) stain was used to detect nuclei. Integrated optical density (IOD) of the specific fluorescent protein was analyzed by the Aipathwell to analyze the expression for the single or double stained fluorescent proteins

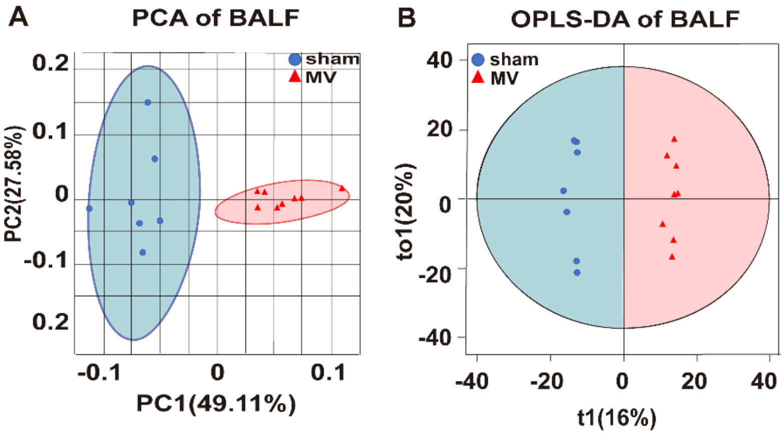

Figure S4. Aerobic glycolysis was associated with MV-induced pulmonary fibrosis. A and B, consistency and reproducibility of bronchoalveolar lavage fluid (BALF) analyzed by principle component analysis (PCA) and orthogonal projections to latent structures-discriminant analysis (OPLS-DA).

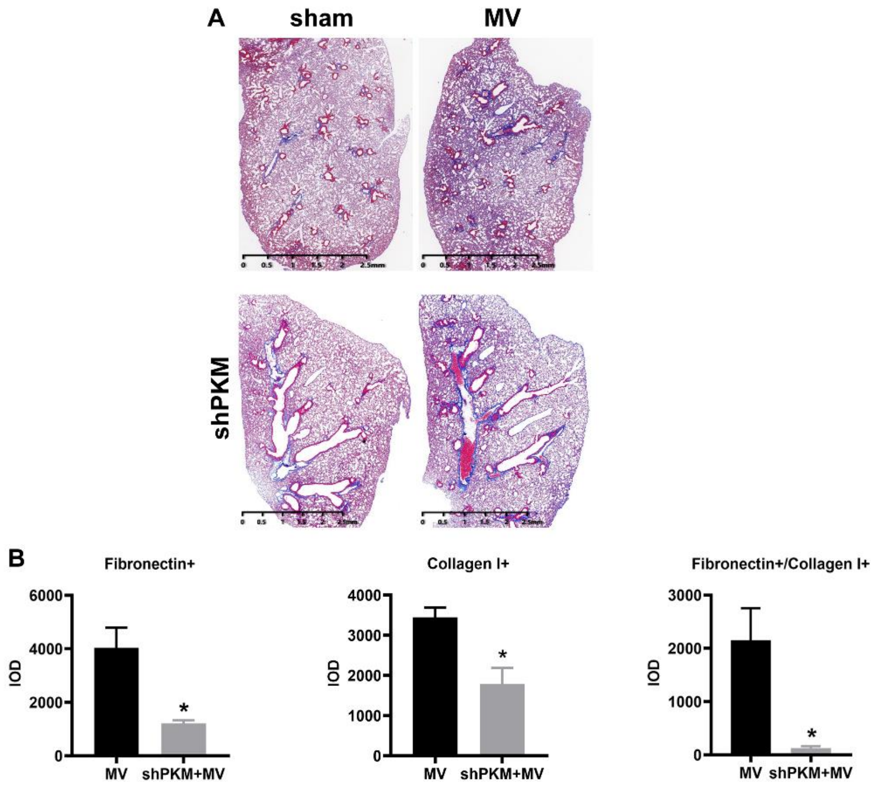

Figure S5. PKM2 knockdown alleviates aerobic glycolysis and MV-induced pulmonary fibrosis. Mice were treated intratracheally with vector-AAV or shPKM-AAV 4 weeks before mechanical ventilation (MV), for the duration of 2 h. Lung tissues were harvested 7 days after intubation. **A,** lung fibrosis was quantified by Masson's trichrome staining. Original magnification × 10, Scale bars correspond to 2.5 mm, n = 6, 3, 6, 5, respectively in each group. **B,** lung tissues were stained with fluorophorelabeled antibodies against fibroblast marker fibronectin and collagen I, 4',6-diamidino-2-phenylindole stain was used to detect nuclei. Integrated optical density (IOD) of the specific fluorescent protein was analyzed by the Aipathwell to analyze the expression for the single or double stained fluorescent proteins.

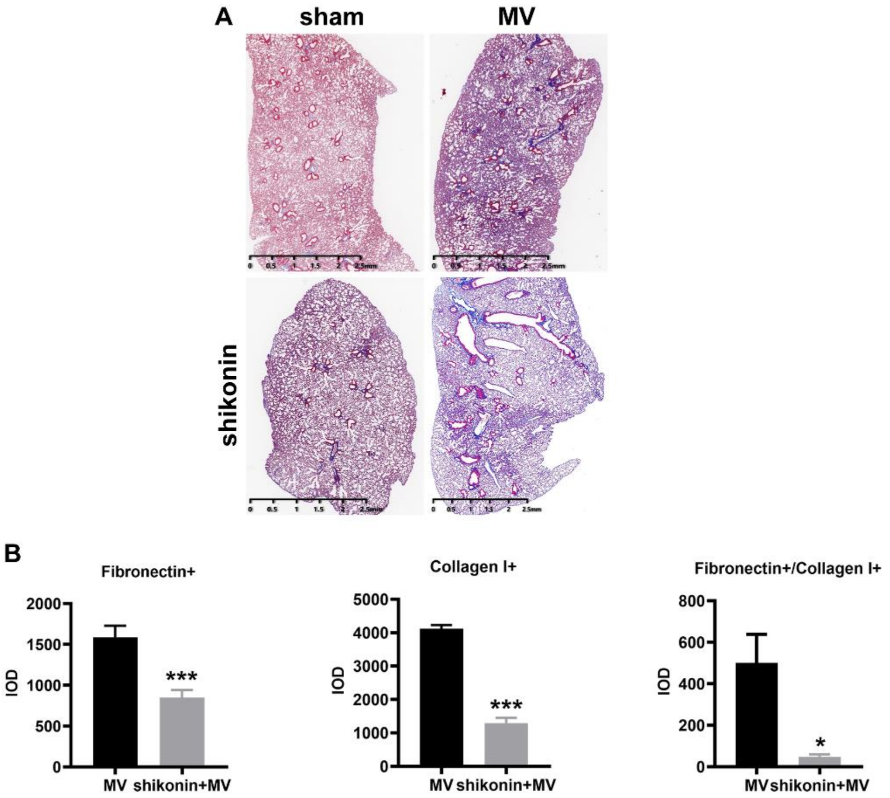

Figure S6. PKM2 inhibitor alleviates aerobic glycolysis and MV-induced pulmonary fibrosis. Mice were treated with PKM2 inhibitor shikonin 10 mg/kg in 100 ul corn oil daily for 3 consecutive days before being subjected to MV for 2 h. Lung tissues were harvested 7 days after intubation. **A,** lung fibrosis was accessed by Masson's trichrome staining, Original magnification × 10, Scale bars correspond to 2.5 mm, n = 6, 3, 6, 3, respectively in each group. **B,** lung tissues were stained with fluorophore-labeled antibodies against fibroblast marker fibronectin and collagen I, 4',6-diamidino-2-phenylindole stain was used to detect nuclei. Integrated optical density (IOD) of the specific fluorescent protein was analyzed by the Aipathwell to analyze the expression for the single or double stained fluorescent proteins.

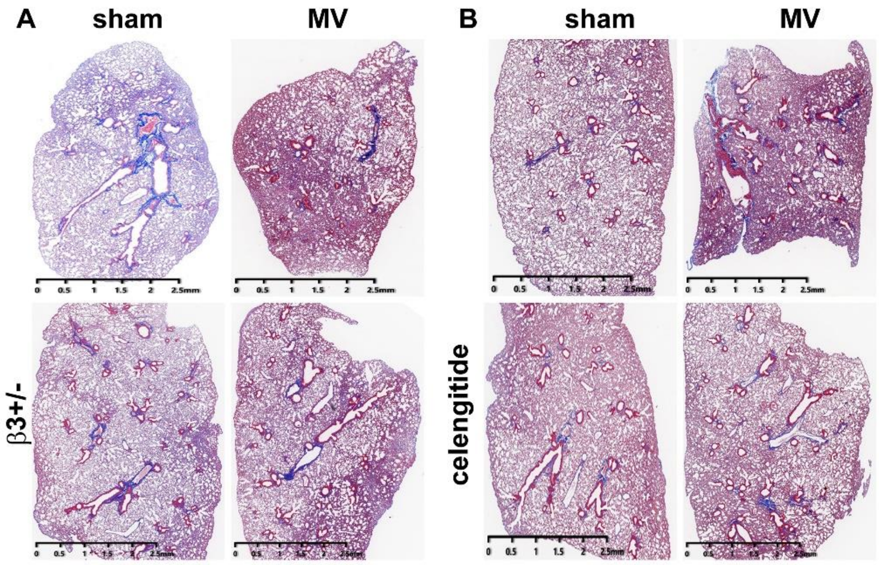

Figure S7. Integrin β3 inhibition ameliorates PKM2–mediated aerobic glycolysis and MV-induced pulmonary fibrosis. **A**, Both wild-type and integrin β3-deficient ($\beta 3^{+/-}$) mice were subjected to mechanical ventilation (MV) for 2 h. Lung tissues were harvested at day 7. Lung fibrosis was quantified by Masson's trichrome staining, original magnification × 10, scale bars correspond to 2.5 mm, n = 6, 5, 6, 6, respectively in each group. **B**, mice were treated intraperitoneally with integrin β3 inhibitor cilengitide 4 mg/kg once a day for 3 consecutive days before being subjected to MV for 2 h. Lung tissues were harvested 7 days after intubation. lung fibrosis was quantified by Masson's trichrome staining, original magnification × 10, scale bars correspond to 2.5 mm, n = 6 per group.

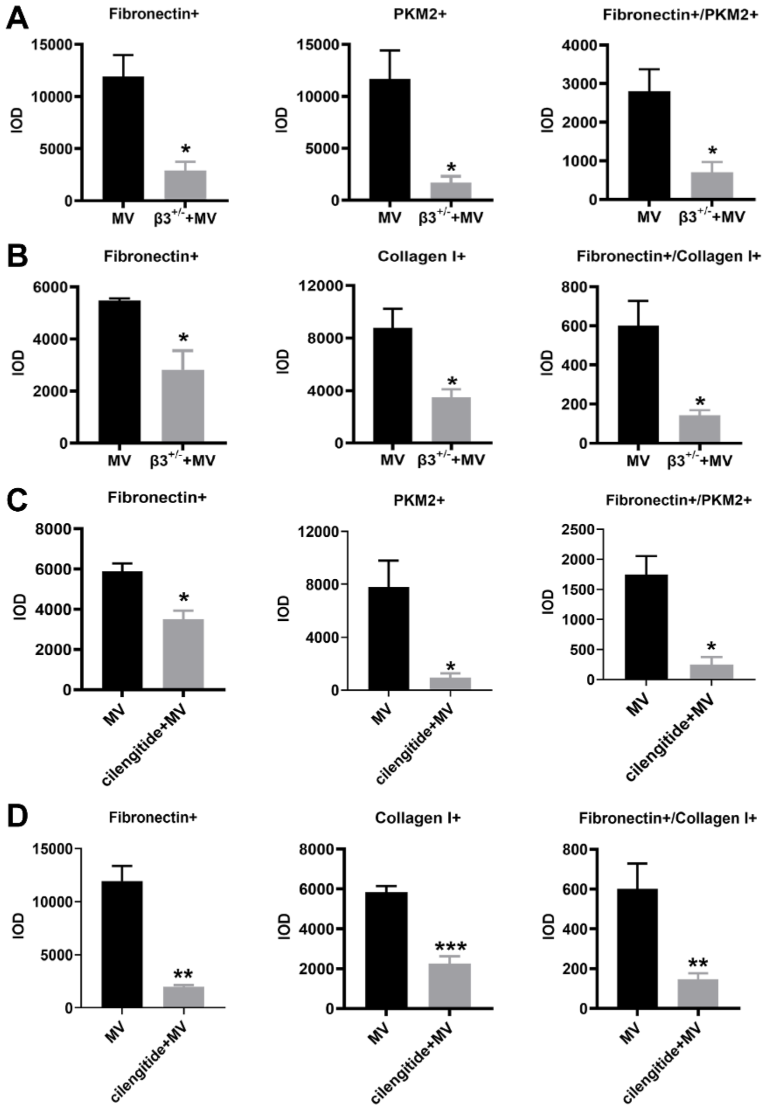

Figure S8. Integrin β3 inhibition ameliorates PKM2–mediated aerobic glycolysis and MV-induced pulmonary fibrosis. A, B, both wild-type and integrin β3-deficient (β3$^{+/-}$) mice were subjected to mechanical ventilation (MV) for 2 h. Lung tissues were harvested at day 7. Lung tissues were stained with fluorophorelabeled antibodies against fibroblast marker fibronectin, pyruvate kinase M2(PKM2)and collagen I, 4',6-diamidino-2- phenylindole (DAPI) stain was used to detect nuclei. **C,D,** mice were treated intraperitoneally with integrin β3 inhibitor cilengitide 4mg/kg once a day for 3 consecutive days before being subjected to MV for 2 h. Lung tissues were harvested 7 days after intubation. Lung tissues were stained with fluorophore-labeled antibodies against fibroblast marker fibronectin, PKM2 and collagen I, DAPI stain was used to detect nuclei. Integrated optical density (IOD) of the specific fluorescent protein was analyzed by the Aipathwell to analyze the expression for the single or double stained fluorescent proteins.

Table S1. CellRanger analysis of cell information. The gene expression quantifed by 10 X scRNA-Seq was mainly based on UMI counts. UMI could distinguish whether a read was a biological repeat or a technical repeat, which could effectively remove the PCR effect. CellRanger removed duplicate UMIs for genes under each barcode and counted the number of unique UMIs that represented the expression of cellular genes.

sampleID	Estimated Number of Cells	Mean Reads per Cell	Median UMI Counts per Cell	Median Genes per Cell	Total Genes Detected
C1	6,278	53,523	5,469	2,205	27,275
C2	7,545	45,264	8,229	2,791	31,879
C3	9,002	36,390	6,642	2,412	31,466
M1	6,125	49,294	6,702	1,561	26,616
M2	8,615	37,877	6,387	2,254	30,360
M3	7,410	44,585	8,043	2,683	31,638
